# Comparative Genomic and Phylogenetic Analysis of Chloroplast Genomes of Hawthorn (*Crataegus* spp.) in Southwest China

**DOI:** 10.3389/fgene.2022.900357

**Published:** 2022-07-04

**Authors:** Xien Wu, Dengli Luo, Yingmin Zhang, Congwei Yang, M. James C. Crabbe, Ticao Zhang, Guodong Li

**Affiliations:** ^1^ College of Chinese Material Medica, Yunnan University of Chinese Medicine, Kunming, China; ^2^ Wolfson College, Oxford University, Oxford, United Kingdom; ^3^ Institute of Biomedical and Environmental Science and Technology, School of Life Sciences, University of Bedfordshire, Luton, United Kingdom; ^4^ School of Life Sciences, Shanxi University, Taiyuan, China

**Keywords:** phylogenetic analysis, comparative analysis, chloroplast genome, hawthorn, *Crataegus* spp.

## Abstract

The hawthorns (*Crataegus* spp.) are widely distributed and famous for their edible and medicinal values. There are ∼18 species and seven varieties of hawthorn in China distributed throughout the country. We now report the chloroplast genome sequences from *C. scabrifolia*, *C. chungtienensis* and *C. oresbia*, from the southwest of China and compare them with the previously released six species in *Crataegus* and four species in Rosaceae. The chloroplast genome structure of *Crataegus* is typical and can be divided into four parts. The genome sizes are between 159,654 and 159,898bp. The three newly sequenced chloroplast genomes encode 132 genes, including 85 protein-coding genes, 37 tRNA genes, and eight rRNA genes. Comparative analysis of the chloroplast genomes revealed six divergent hotspot regions, including *ndhA*, *rps16*-*trnQ*-UUG, *ndhF*-*rpl32*, *rps16*-*psbK*, *trnR*-UCU-*atpA* and *rpl32*-*trnL*-UAG. According to the correlation and co-occurrence analysis of repeats with indels and SNPs, the relationship between them cannot be ignored. The phylogenetic tree constructed based on the complete chloroplast genome and intergenic region sequences indicated that *C. scabrifolia* has a different origin from *C. chungtienensis* and *C. oresbia*. We support the placement of *C. hupehensis, C. cuneata*, *C. scabrifolia* in *C.* subg. *Crataegus* and *C. kansuensis*, *C. oresbia*, *C. kansuensis* in *C.* subg. *Sanguineae*. In addition, based on the morphology, geographic distribution and phylogenetic relationships of *C. chungtienensis* and *C. oresbia*, we speculate that these two species may be the same species. In conclusion, this study has enriched the chloroplast genome resources of *Crataegus* and provided valuable information for the phylogeny and species identification of this genus.

## 1 Introduction

Chloroplasts are semi-independent organelles derived from blue-green algae through endosymbiosis and play an important role in the transfer and expression of genetic material. (Margulis, 1976; Wolfe et al., 1987; Bremer et al., 2002). The chloroplast genome of angiosperms is highly conserved and consists of four parts, including two inverted repeats (IRa and IRb), a large single copy region (LSC) and a small single copy region (SSC) (Palmer, 1991). Before the development of sequencing technology, the complete chloroplast genome required a lot of manpower and material resources. Therefore, some genome segments for phylogenetic analysis were identified before the entire genome sequence was available (Shaw et al., 2005; Dong et al., 2012). These segments contain significant species-level genetic variability and divergence to distinguish different species (Kress and Erickson, 2008). At the same time, these segments are highly conserved in plant evolution, and this conservatism and uniparental heritance make it possible to compare phylogenetic relationships at various taxonomic levels (Katayama and Uematsu, 2005; Kress and Erickson, 2008). At present, genome segments commonly used to study the phylogenetic relationship of species include gene regions (*rpoC1*, *rpoB*, *matK*, *rbcL*, *rps16*, *rpl16*) and intergenic regions (*atpF-atpH*, *trnH-psbA*, *psbK-psbI*, *trnG-trnS*, *trnH-rpl2*, *rpl20-rps12*, *trnC-ycf6*, *atpB-rbcL*, *trnL-trnF*) (Hollingsworth et al., 2009; Lo and Donoghue, 2009; Lo et al., 2009; Zarrei et al., 2015). Since the *Nicotiana tabacum* plastid genome sequence was published (Shinozaki et al., 1986), the chloroplast genomes of 3,452 plants have been published on NCBI (27 October 2019), and using chloroplast genomes for DNA barcoding is an important developmental tool (Yu et al., 2021). Thus, the chloroplast genome has always been important for plant identification, classification and phylogeny (Zhao et al., 2018; Biju et al., 2019; Nguyen et al., 2021).

The hawthorns (*Crataegus* spp.) are a genus which has been used as edible, medicinal, ornamental, and horticultural plants. Hawthorn is very popular as a sour fruit, and its fruit has been processed into jam, candy and other foods, with high economic value. Hawthorn is also an important traditional Chinese medicine which helps digest food, enhances stomach function, lowers blood pressure and blood lipids (Chinese Pharmacopoeia Commission 2020). In addition, modern pharmacological studies show that hawthorn can promote blood circulation, have antihyperlipidemic, hepatoprotective, antioxidant, antiaging, antitumor, and antibacterial properties (Jurikova et al., 2012; Zhu et al., 2015; Dong et al., 2017).

*Crataegus* contains more than 250 species (Evans and Campbell, 2002), in which ∼18 species and 7 varieties are distributed throughout China (Gu and Spongberg, 2003). There are some unique hawthorn resources in southwest China, however, current chloroplast genome and phylogenetic studies of *Crataegus* only contain a few samples from the area. Therefore, this study assembled three *Crataegus* chloroplast genomes from southwest China, including *Crataegus scabrifolia* (Franch.) Rehder, *Crataegus chtsngtienensis* W.W. Smith*, Crataegus. oresbia* W. W. Smith, and compared them with six *Crataegus* (*Crataegus pinnatifida* var. *major* N.E.Br., *Crataegus pinnatifida* Bunge, *Crataegus hupehensis* Sarg., *Crataegus kansuensis* Wils., *Crataegus cuneata* Sieb. et Zucc., *Crataegus marshallii* Eggl.) and four other species of Rosaceae (*Eriobotrya salwinensis* Hand.-Mazz. (Liu et al., 2019b), *Malus × micromalus* Makino (Hu et al., 2017), *Spiraea mongolica* Maxim. (Ma et al., 2021), and *Rubus phoenicolasius* Maxim. (Zhang G et al., 2021). We conducted a comprehensive analysis of the *Crataegus* chloroplast genomes, including basic genome structure, analysis of codon usage, repetitive structure characteristics, comparative genomic and phylogenetic analyses of the chloroplast genomes of *Crataegus*. In addition, phylogenetic trees were constructed using five intergenic regions to explore the subgenus classification of 8 hawthorn species native to China. Through these studies we will enrich the information of hawthorn chloroplast genomes and explore the relationships between species within *Crataegus* and between *Crataegus* with other species of Rosaceae.

## 2 Materials and Methods

### 2.1 Plant and DNA Sources

Healthy fresh leaves of *C. scabrifolia* were obtained from the Kunming Institute of Botany, Chinese Academy of Sciences and healthy fresh leaves of *C. chungtienensis* and *C. oresbia* were obtained from the Shangri-la Alpine Botanical Garden (Supplementary Figure S1). The voucher specimens *C. scabrifolia* (YUNCM5301260363), *C. chungtienensis* (YUNCM2021051701) and *C. oresbia* (YUNCM2021051702) were deposited in the Herbarium of Yunnan University of Chinese Medicine (YUNCM). DNA was extracted from the leaves of the above three plants for each species using the Plant Genomic DNA Kit and the DNA concentration and quality were detected by a NanoDrop 2000 spectrophotometer and agarose gel electrophoresis.

### 2.2 DNA Sequencing, Assembly, and Annotation

The obtained total DNA was sequenced on an Illumina Hiseq 4000. The chloroplast genome sequences were assembled by NOVOPlasty v2.7.2 (Wyman et al., 2004); the size of k-mers was 39. The chloroplast genomes were annotated, compared, checked and corrected with Geneious Prime®2020.1.1 (Greiner et al., 2019). Transfer RNAs (tRNAs) were annotated by tRNAscan-SE software (Lohse et al., 2007). The genes, introns and the boundaries of coding regions were compared with reference sequences. Finally, the OGDRAW tool (Greiner et al., 2019) was used to display a circular chloroplast genome map.

### 2.3 Structural Analyses

Total chloroplast genome length, length of LSC, SSC and IR, GC content, number of genes and frequency of amino acids were counted by Geneious Prime®2020.1.1 (Greiner et al., 2019). Codon usage bias analysis was performed using MEGA X (Beier et al., 2017) software. The online MISA (Beier et al., 2017) tool was used to detect simple sequence repeats. The minimum repeat number of mononucleotides, dinucleotides, trinucleotides, tetranucleotides, pentanucleotides, and hexanucleotides was set to 10, 5, 4, 3, 3, and 3, respectively. The repeat sequences (forward, palindromic, reverse, and complement repeats) were detected using REPuter (Kurtz et al., 2001) with the minimum repeat size 30 bp, and the Hamming Distance 3.

Using *C. chungtienensis* as the reference sequence, the indels and SNPs of the other five chloroplast genomes (*C. scabrifoli*, *E. salwinensis*, *M. micromalus*, *S. mongolica*, *R. phoenicolasius*) were analyzed by snippy software (Seemann, 2015) (Supplementary Table S1), as used previously (Abdullah et al., 2020; Liu et al., 2020). Oligonucleotide repeats in the reference genome were counted using REPuter (Kurtz et al., 2001). The minimal repeat sizewas set to 14 bp, and there was no mismatch between the two copies of the repeat sequence. The repeats are the sum of oligonucleotide repeats and SSRs. Using *C. chungtienensis* as the coordinate reference sequence, other five sequences were aligned using Geneious Prime® 2020.1.1 software (Greiner et al., 2019) and the co-occurrence of repeats with SNPs and indels was counted. Then, indels, SNPs and repeats were counted within non-overlapping 150 bp windows of the chloroplast genome (Supplementary Table S2). Spearman’s rho correlation was performed using SPSS software version 26.0 to calculate the degree of association between indels, SNPs, and repeats. The strength levels of the correlations were classified as follows: very weak (0.1–0.19), weak (0.20–0.29), moderate (0.30–0.39), strong (0.4–0.69), very strong (0.70–0.99) and perfect (1.0). The probability (p) of significance of the correlation was tested at α-level of 0.01.

### 2.4 Comparative Genomic Analysis

The whole-genome alignment of *Crataegus* and relatives was compared by the mVISTA program in Shuffle-LAGAN mode (Beier et al., 2017). On the basis of annotations, the chloroplast genome borders between the IR, LSC, and SSC were analyzed and the schematic diagram was drawn with IRscope (Amiryousefi et al., 2018). Nucleotide variability (Pi) was computed by DnaSP v5.10. Then the divergence hotspot values of *Crataegus* chloroplast genomes were calculated by sliding window analysis; the window length was set to 600 bp and the step length was set to 200 bp (Librado and Rozas, 2009).

### 2.5 Phylogenetic Analysis

We conducted a phylogenetic analysis of Rosaceae, including *C. scabrifolia, C chungtienensis*, and *C. oresbia*. The other 47 species of Rosaceae and the outgroups *Broussonetia kwaii* (Moraceae) and *Morus alba* (Moraceae) were downloaded from NCBI (Supplementary Table S3). The Phylogenetic trees of complete chloroplast genomes were constructed using the maximum likelihood (ML) method and the Bayesian inference (BI) method. ML analysis was conducted by IQ-TREE (version 2.1.3) in Phylosuite (Bennetzen et al., 2005; Zhang D et al., 2020) software with a GTR + F + I substitution model and 1,000 bootstrap replicates. The Bayesian inference (BI) tree was implemented in MrBayes in Phylosuite (Zhang D et al., 2020) and was run for two million generations in total. Based on the Markov chain Monte Carlo (MCMC) algorithm (Gascuel, 1997), the best-fitting GTR + F + I substitution model was determined with sampling after every 1,000 generations. When the value of the average standard deviation of split frequencies was less than 0.01 we stopped running. Then we discarded less than 25% of the aging samples and constructed a consistent tree according to the remaining samples.

In addition, we selected ten diploid species from five *Crataegus* subgenus, including *C*. subg. *Americanae* (*C. crus-galli*, *C. punctata* and *C. trifloral*), *C*. subg. *Crataegus* (*C. pentagyna*), *C*. subg. *Sanguineae* (*C. saligna*, *C. suksdorfii*, *C. nigra* and *C. wilsonii*), *C*. subg. *Mespilus* (*C. germanica*) and *C.* subg. *Brevispinae* (*C. brachyacantha*) (Phipps et al., 1990; Zarrei et al., 2015; Ufimov and Dickinson, 2020; Liston et al., 2021), and downloaded their intergenic regions sequences from NCBI (*atpF*-*atpH*, *trnL-trnF*, *atpB-rbcL*, *rpl20-rps12* and *rpl2-trnH*). An outgroup (*Amelanchier alnifolia* (a)) was also downloaded from NCBI. Using Geneious Prime® 2020.1.1 software (Greiner et al., 2019), identical intergenic regions were extracted from the complete chloroplast genomes of nine hawthorns and outgroups (*Amelanchier pallida*, *Amelanchier alnifolia* (b), *Eriobotrya japonica* and *Eriobotrya salwinensis*) (Supplementary Table S4). The phylogenetic trees were constructed by the concatenated sequence of five intergenic regions, and the methods for constructing the phylogenetic tree were the same as above.

## 3 Results

### 3.1 General Features of *Crataegus*

**FIGURE 1:**
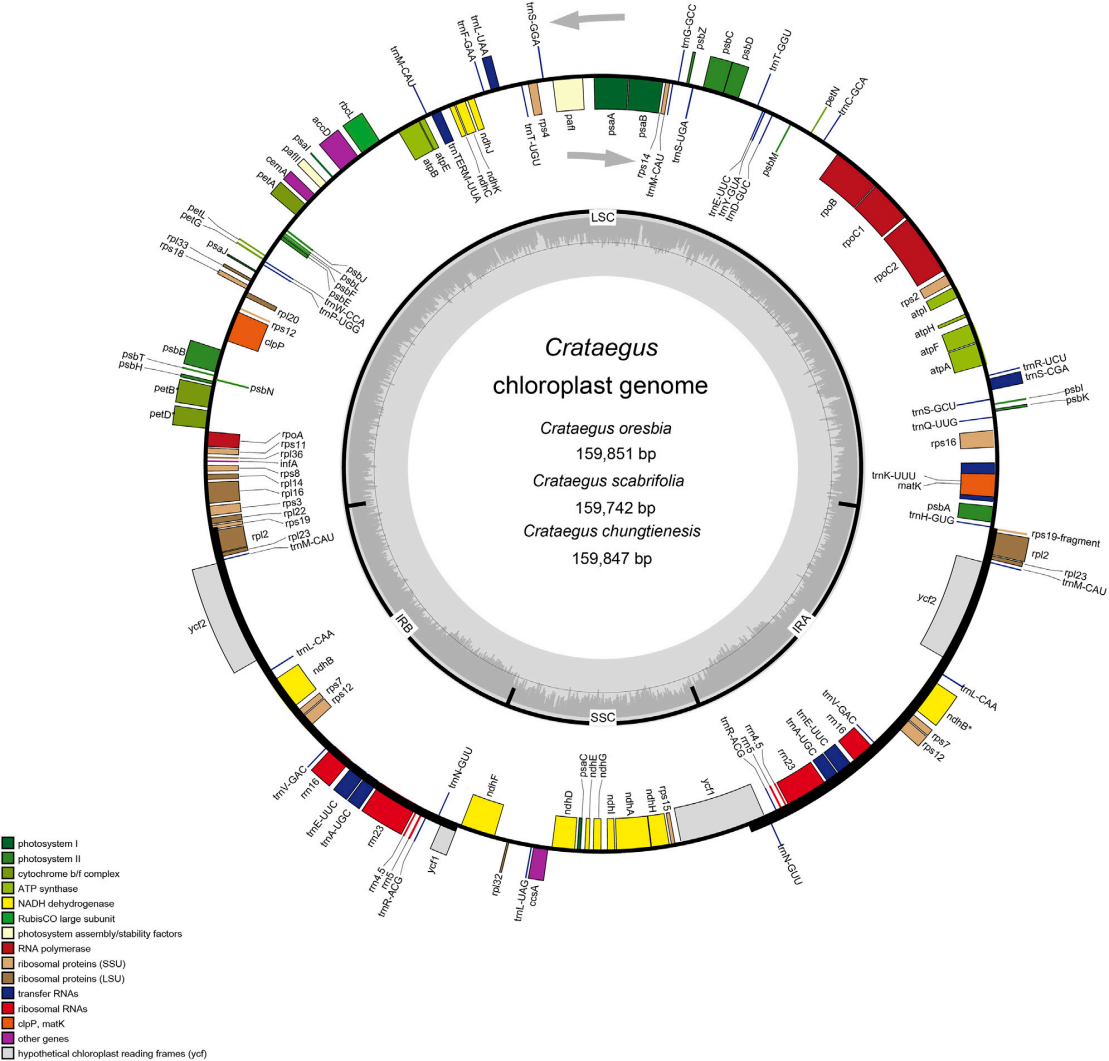
Chloroplast genome maps of *Crataegus* (*C. oresbia*, *C. scabrifolia*, *C. chungtienesis*). In the diagram, different colors indicate genes with different functions. The genes inside circles are transcribed clockwise and genes outside circles are transcribed counterclockwise. Two inverted repeats (IRa and IRb), a large single copy region (LSC) and small single copy region (SSC) regions are shown in the inner circles. The light gray inner circles indicate A/T content and the dark gray circles indicate G/C content.

All of these nine chloroplast genomes of *Crataegus* have a typical circular tetramerous structure, including two inverted repeats (IRs), a large single copy (LSC) and a small single copy (SSC) (Figure 1). The chloroplast genomes size varied from 159,654 bp (*C. pinnatifida* var. *major*) to 159,898 bp (*C. pinnatifida*). The LSC size was from 87,599 bp (*C. pinnatifida*) to 87,857 bp (*C. scabrifolia*), the SSC size from 19,138 bp (*C. pinnatifida* var. *major*) to 19,263 bp (*C. oresbia*), and the IR size from 26,384 bp (*C. cuneata*) to 26,540 bp (*C. pinnatifida*). The overall GC content of the chloroplast genomes was 36.6% (Table 1).

**TABLE 1:** Comparison of chloroplast genome features of the nine species of *Crataegus* and four other genera of Rosaceae (*Eriobotrya*, *Malus*, *Rubus*, and *Spiraea*).

Species	Size (bp)	LSC (bp)	SSC (bp)	IR (bp)	Genes number	GC (%)	Reference
*C. oresbia*	159,851	87,819	19,263	26,384	132	36.6	This study
*C. scabrifolia*	159,742	87,819	19,218	26,383	132	36.6	This study
*C. chungtienesis*	159,847	87,814	19,263	26,384	132	36.6	This study
*C. marshallii*	159,660	87,712	19,231	26,358	132	36.6	Liu et al. (2019a)
*C. pinnatifida*	159,898	87,599	19,218	26,540	129	36.6	He et al. (2020)
*C. cuneata*	159,730	87,778	19,183	26.384	129	36.6	
*C. kansuensis*	159,865	87,815	19,231	26,384	132	36.6	Zhang X et al. (2020)
*C. hupehensis*	159,766	87,852	19,143	26,385	132	36.6	Hu et al. (2021b)
*C. Pinnatifida* var. *major*	159,654	87,747	19,138	26,384	131	36.6	Wu et al. (2021)
*E. salwinensis*	159,488	87,380	19,290	26,384	132	36.7	Liu et al. (2019b)
*M. micromalus*	159,834	87,950	19,176	26,409	129	36.6	Hu et al. (2017)
*S. mongolica*	155,949	84,375	18,894	26,354	131	36.7	Ma et al. (2021)
*R. phoenicolasius*	155,144	84,818	18,580	25,937	130	37.3	Zhang G et al. (2021)

A total of 132 genes were detected in the three newly sequenced species (*C. scabrifolia, C chungtienensis, C. oresbia*), including 85 protein-coding genes, 37 tRNA genes, and eight rRNA genes. The LSC region contained 63 protein-coding and 22 tRNA genes, while the SSC region contained 12 protein-coding genes and one tRNA gene. Ten protein-coding genes, 14 tRNA coding genes and eight rRNA coding genes were located within IRs. In the chloroplast genomes of the three species of *Crataegus*, 18 genes contained introns, of which two genes (*ycf3* and *clpP*) contained two introns, whereas the remaining 16 (*trnk*-UUU, *rps16*, *trnG*-GCC, *atpF*, rpoC1, *trnL*-UAA, *trnV*-UAC, *rpl2*, *ndhB*, *rps12*, *trnI*-GAU, *trnA*-UGC, *ndhA*, *petB*, *petD*, *rpl16*) genes contained one intron (Table 2). In addition, we counted the intergenic regions of nine hawthorn chloroplast genomes (Supplementary Table S5).

**TABLE 2:** Genes in the chloroplast genome of *Crataegus chungtienesis.*

Gene Group	Gene name
Photosystem I	*psaA*, *psaB*, *psaC*, *psaI*, *psaJ*
Photosystem II	*psbA*, *psbB*, *psbC*, *psbD*, *psbE*, *psbF*, *psbH*, *psbI*, *psbJ*, *psbK*, *psbL*, *psbN*, *psbT*, *psbZ*
NAD (P) H oxidoreductase	*ndhA* ^a^, *ndhB* ^a,c^, *ndhC*, *ndhD*, *ndhE*, *ndhF*, *ndhG*, *ndhH*, *ndhI*, *ndhJ*, *ndhK*
Cytochrome b6/f complex	*petA*, *petB* ^a^, *petD* ^a^, *petG*, *petL*, *petN*
ATP synthase	*atpA*, *atpB*, *atpE*, *atpF* ^a^, *atpH*, *atpI*
Rubisco	*rbcL*
Large subunit of ribosomal	*rpl2* ^ac^, *rpl14*, *rpl16*, *rpl20*, *rpl22*, *rpl23* ^c^, *rpl32*, *rpl33*, *rpl36*
Small subunit of ribosomal	*rps2*, *rps3*, *rps4*, *rps7* ^c^, *rps8*, *rps11*, *rps12* ^*a,*b,c^, *rps14*, *rps15*, *rps16* ^a^, *rps18*, *rps19* ^c^
DNA dependent RNA polymerase	*rpoA*, *rpoB*, *rpoC1* ^a^, *rpoC2*
rRNA genes	*rrn4.5* ^c^, *rrn5* ^c^, *rrn16* ^c^, *rrn23* ^c^
tRNA genea	*trnA-*UGC^a,c^, *trnC-*GCA, *trnD-*GUC, *trnE-*UUC, *trnF-*GAA, *trnfM-*CAU, *trnG-*GCC^a^, *trnG-*UCC^a^, *trnH-*GUG, *trnI-*CAU^c^, *trnI-*GAU^a,c^, *trnK-*UUU^a^, *trnL-*CAA^c^, *trnL-*UAA^a^, *trnL-*UAG, *trnfM-*CAU, *trnN-*GUU^c^, *trnP-*UGG, *trnQ-*UUG, *trnR-*ACG^c^, *trnR-*UCU, *trnS-*GCU, *trnS-*GGA, *trnS-*UGA, *trnT-*GGU, *trnT-*UGU, *trnV-*GAC^c^, *trnV-*UAC^a^, *trnW-*CCA, *trnY-*GUA
Maturase	*matK*
Protease	*clpP* ^b^
Envelop membrane protein	*cemA*
Subunit of acetyl-CoA	*accD*
c-type cytochrome synthesis gene	*ccsA*
Translational	*infA*
Conserved hypothetical chloroplast ORF	*ycf1* ^c^, *ycf2* ^c^, *ycf3* ^b^, *ycf*4

Gene^a^, Gene with one intron; Gene^b^, Gene with two introns; Gene^c^, Number of copies of multi-copy genes.

### 3.2 Amino Acid Abundance and Codon Usage

**FIGURE 2:**
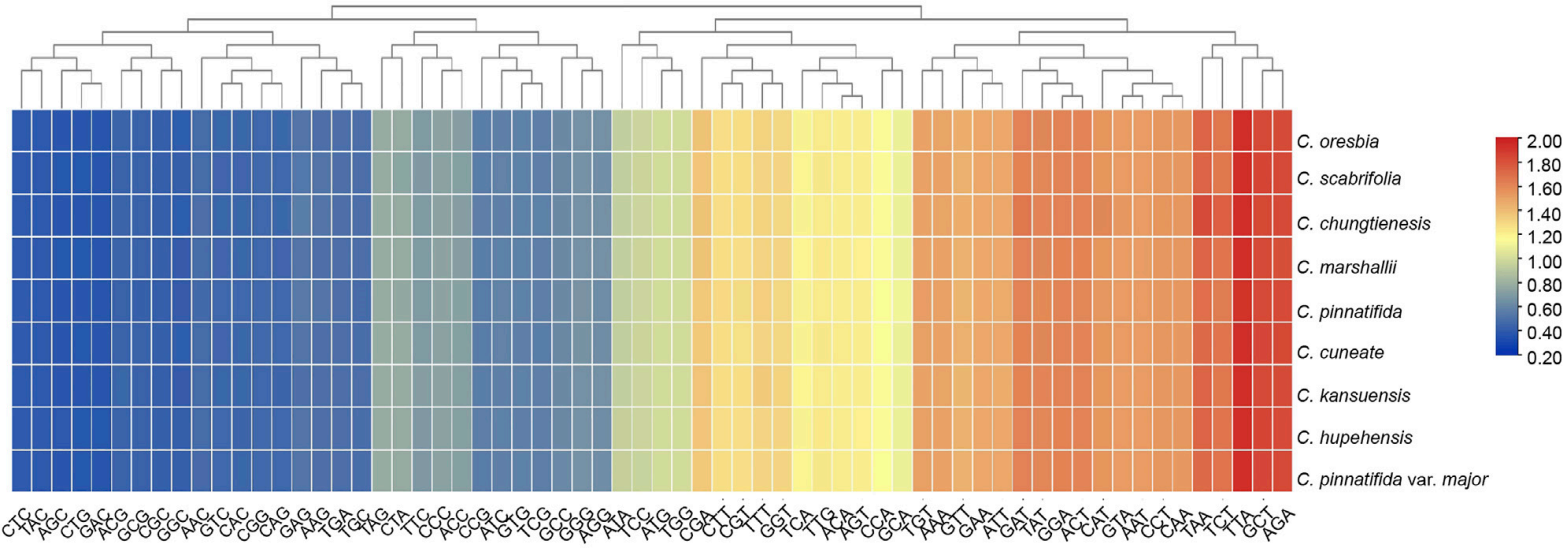
Heatmap analysis of relative synonymous codon usage (RSCU) values among the 9 species of Crataegus.

A total of 61 codons were found in the chloroplast protein coding genes of the nine species. All protein-coding genes consisted of 25,058 (*C. pinnatifida*)—26,218 (*C. chungtienensis*) codons. Among the amino acids encoded by these codons, Leucine (Leu) was the most abundant, followed by Isoleucine (Ile) while Cysteine (Cys) was the least common amino acid (Supplementary Figure S2). According to the Relative Synonymous Codon Usage (RSCU) value, the synonymous codon preference was divided into three grades: high preference, RSCU > 1.0; no preference, RSCU = 1.0; low preference, RSCU < 1.0. The result showed that there were 31 codons with RSCU values > 1.0; out of the above 31 codons, 29 codons were A/T-ending codons. Of those, 38.71% were A-ending codons and 51.61% were T-ending codons. In contrast, there were 33 codons with RSCU values < 1.0, 29 of which ended in G/C. Among them, 48.39% were C-ending codons and 45.16% were G-ending codons. Stop codon usage was found to be biased toward TAA (RSCU > 1.0) (Figure 2).

### 3.3 SSRs and Long Repeats Analyses

A total of 880 SSRs were identified from chloroplast genomes of nine species in *Crataegus* using the MISA software tool. In detail, 98 SSRs were detected in *C. oresbia*, 97 in *C. scabrifolia*, 98 in *C chungtienensis,* 96 in *C. marshallii*, 92 in *C. Pinnatifida*, 96 in *C. cuneata*, 103 in *C. kansuensis*, 100 in *C. hupehensis*, and 99 in *C. pinnatifida* var. *major*. Among the detected mono-, di-, tri-, tetra-, penta-, and hexa-nucleotide SSRs, the single-nucleotide SSRs were the most (ranged from 74 to 63), which accounted for 69.55% of the total number of SSRs (Supplementary Figure S3A). In addition, the di-nucleotide and tetra-nucleotide accounted for the total number of SSRs at 21.25 and 4.89%, respectively. A/T repeats were the most common of mononucleotides (65.61%), while AT/TA repeats were the majority of dinucleotide repeat sequences (20.20%) and tetranucleotide AAAT/TTTA (4.88%) repeats were the third most abundant SSRs types (Supplementary Figure S3B).

More complex and longer repeats may play an important role in the genome. 439 long repeat sequences were identified from the nine species of *Crataegus* chloroplast genomes using the REPuter. 47 long repeats were detected in *C. marshallii*, and 49 long repeats were detected in the remaining 8 chloroplast genomes, including 196 forward repeats, 192 palindromic repeats, 39 reverse repeats, and 12 complementary repeats (Supplementary Figure S3C). The length of these repeat sequences ranged from 30 to 70 bp (Supplementary Figure S3D).

### 3.4 Comparative Analysis of Chloroplast Genomes

**FIGURE 3:**
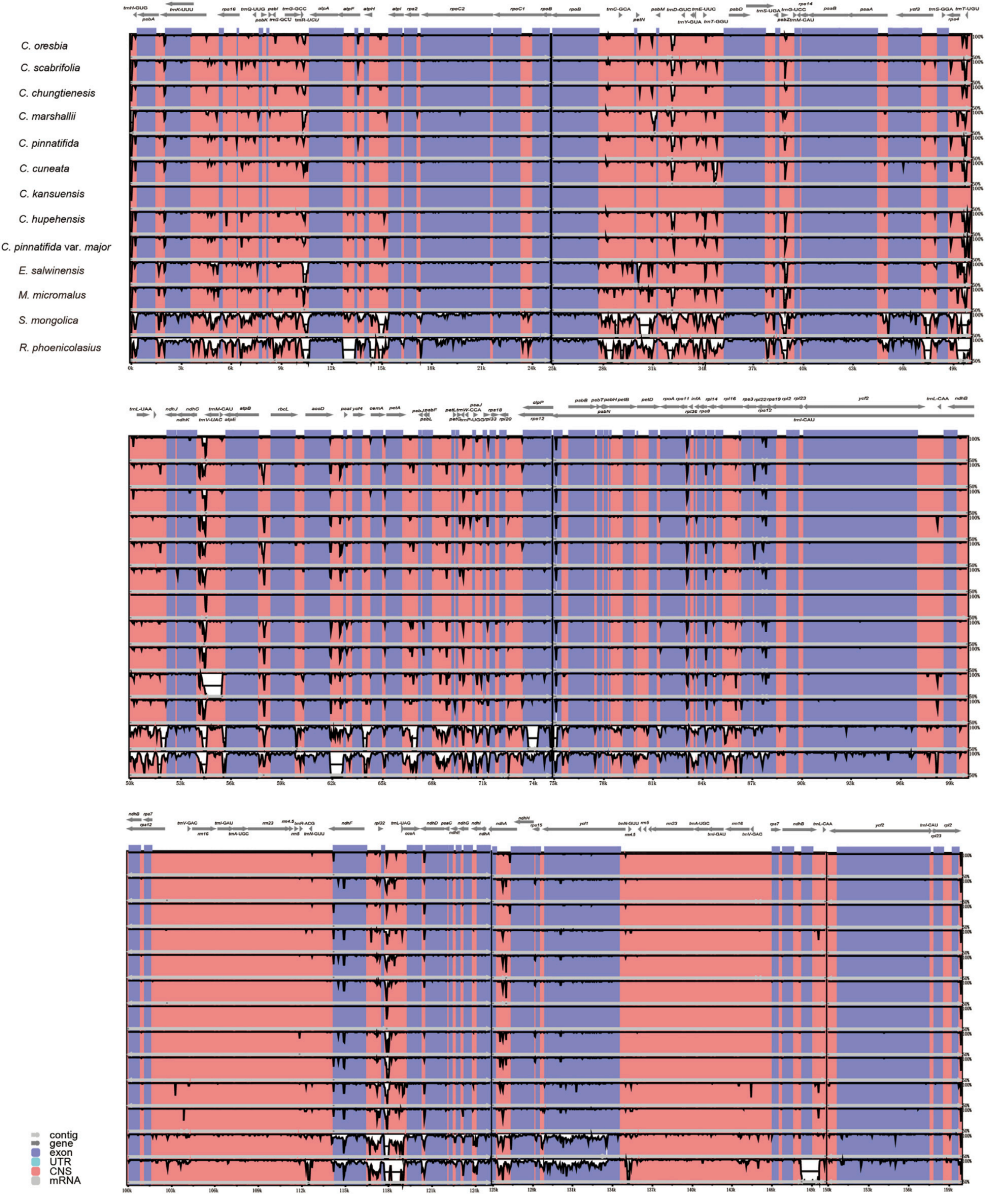
Complete chloroplast genome comparison of 13 species of Rosaceae. Gray arrows indicate the direction of the gene. The dark blue regions represent exons. Pink regions represent noncoding sequences (CNS), and white peaks represent genomic differences. The Y-axis represents the percentage, from 50 to 100%.

Using the annotated *C. kansuensis* chloroplast genome as a reference, our results showed that the nine *Crataegus* chloroplast genomes were relatively conserved and the IR region was more conserved than the LSC and SSC regions. The highly divergent regions were mainly located in the intergenic regions, such as *trnH*-GUG-*trnT*-UGU, *trnR*-UCU-*atpA*, *petN*-*psbM*, *psbM*-*trnD*-GUC, *trnY*-GUA-*psbD*, *ndhC*-*trnV*-UAC, *atpB*-*rbcl*, *accD*-*ycf4*, *rpl32*-*trnL*-UAG (Figure 3). As expected, the other four species of Rosaceae were significantly different from *Crataegus* in the noncoding regions, especially *S. mongolica* and *R. phoenicolasius*, which are more distantly related to *Crataegus*.

**FIGURE 4:**
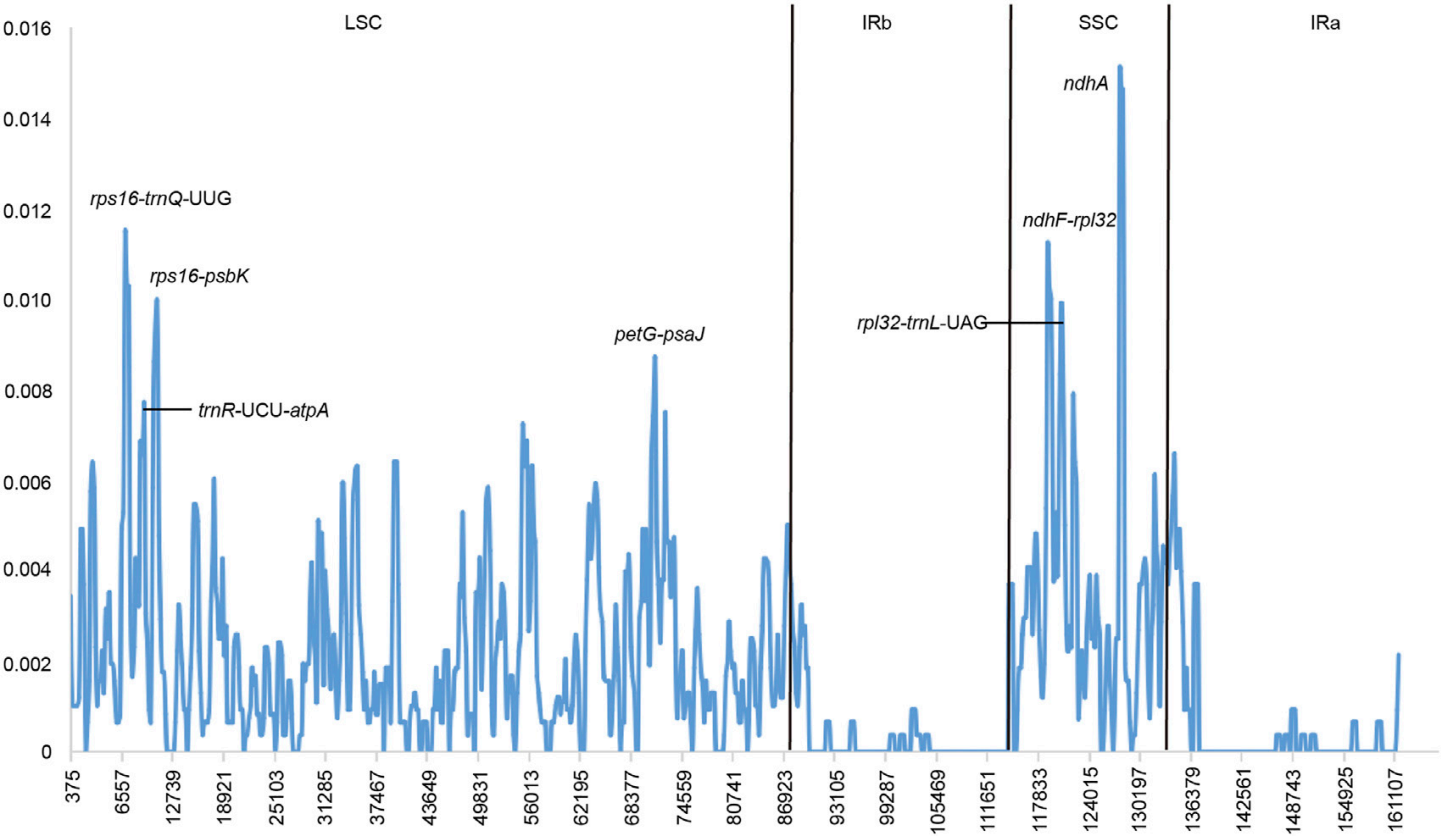
Sliding window analysis based on the cp genomes of 9 *Crataegus* species.

We used DNAsp software to examine nucleotide diversity (Pi), and the divergent hotspot regions in the nine species of *Crataegus* were determined by comparison of the Pi values. The analysis indicated that the average Pi was 0.00175. Furthermore, five highly variable regions (*rps16*-*trnQ*-UUG, *ndhF*-*rpl32*, *rps16*-*psbK*, *trnR*-UCU-*atpA*, *rpl32*-*trnL*-UAG) and a protein-coding gene (*ndhA*) were screened. Among these divergent hotspots, the *ndhA* (Pi = 0.01509) region had the highest Pi values, followed by the *rps16*-*trnQ*-UUG (pi = 0.01148), *ndhF*-*rpl32* (pi = 0.01120), *rps16*-*psbK* (pi = 0.01028), *trnR*-UCU-*atpA* (pi = 0.00995) and *rpl32*-*trnL*-UAG (pi = 0.00991) (Figure 4).

**FIGURE 5:**
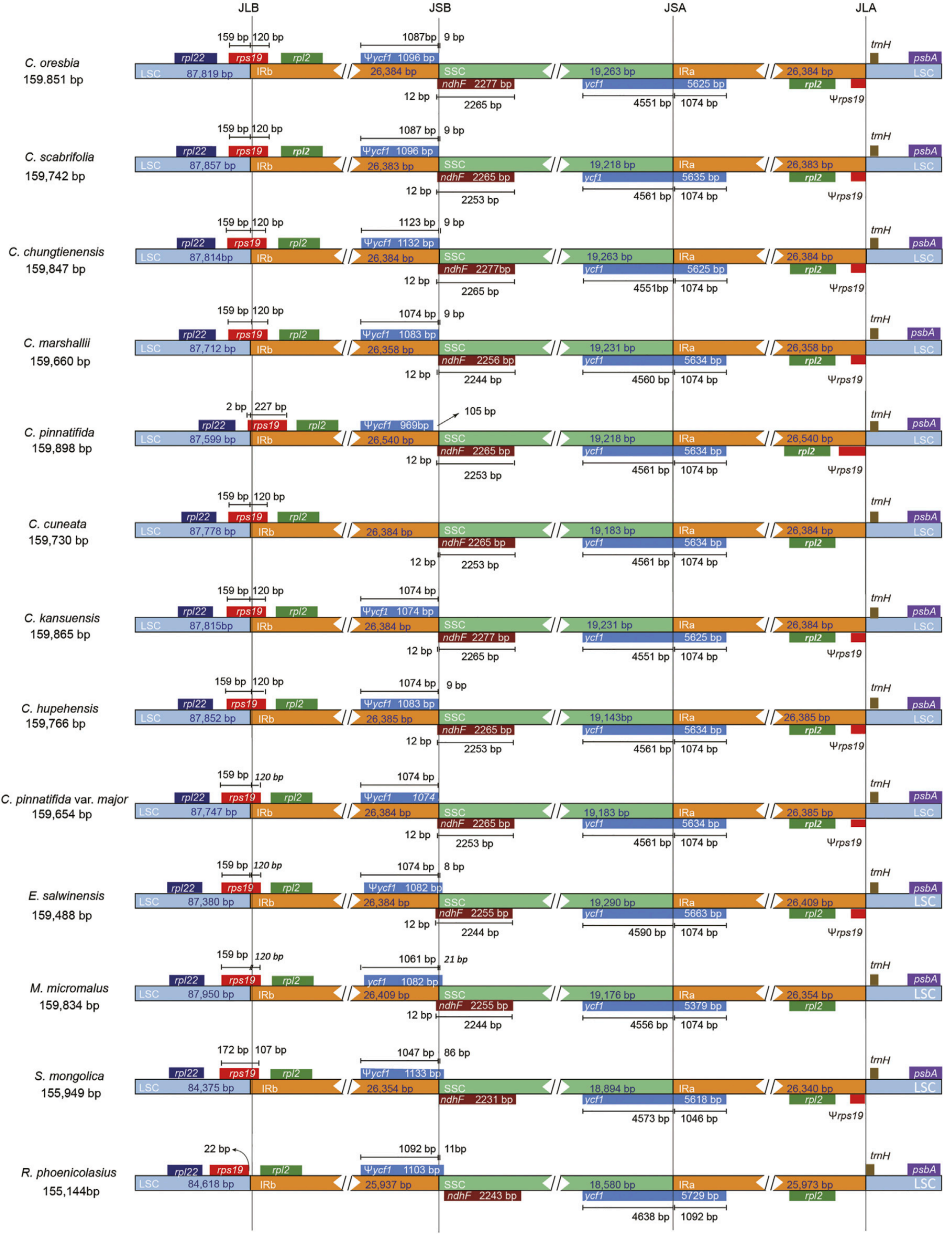
Comparison of the borders of the LSC, SSC, and IR regions among 9 *Crataegus* chloroplast genomes and four species of Rosaceae.

We compared the chloroplast genomes of hawthorns with other four species of Rosaceae (*E. salwinensis*, *M. micromalus*, *S. mongolica*, *R. phoenicolasius*). Their chloroplast genome structures were highly conserved, but there were some differences (Figure 5). The genes *rpl22*, *rps19*, *rpl2*, *ycf1*, *ndhF*, *trnH*-GUG, and *psbA* were present at the junction of the IRb/LSC (JLB), SSC/IRb (JSB), IRa/SSC (JSA), and LSC/IRa (JLA) borders. In addition, *rps19* (except *C. pinnatifida*) was mainly located in the LSC region, *ycf1* was mainly located in the SSC region, *ndhF* was mainly located in the SSC region. Most of the sequence variations were distributed in LSC and SSC regions while IR regions exhibited relatively few sequence variations (Khakhlova and Bock, 2006). Even so, the contractions and expansions at the borders of IR regions are common evolutionary events and may cause size variations of chloroplast genomes (Biju et al., 2019; Li et al., 2019; Luo et al., 2021). The IR regions of *Rubus* were contracted, and *rps19* only appeared in the LSC region, so the gene is not copied, resulting in a shorter gene length of *R. phoenicolasius*. In contrast, the IR of *C. pinnatifida* was further expanded, *rps19* was mainly located in the IRb region, so the gene is almost completely replicated in the IR region, and the gene length of *C. pinnatifida* is also longer compared with other species.

### 3.5 Phylogenetic Analyses

**FIGURE 6:**
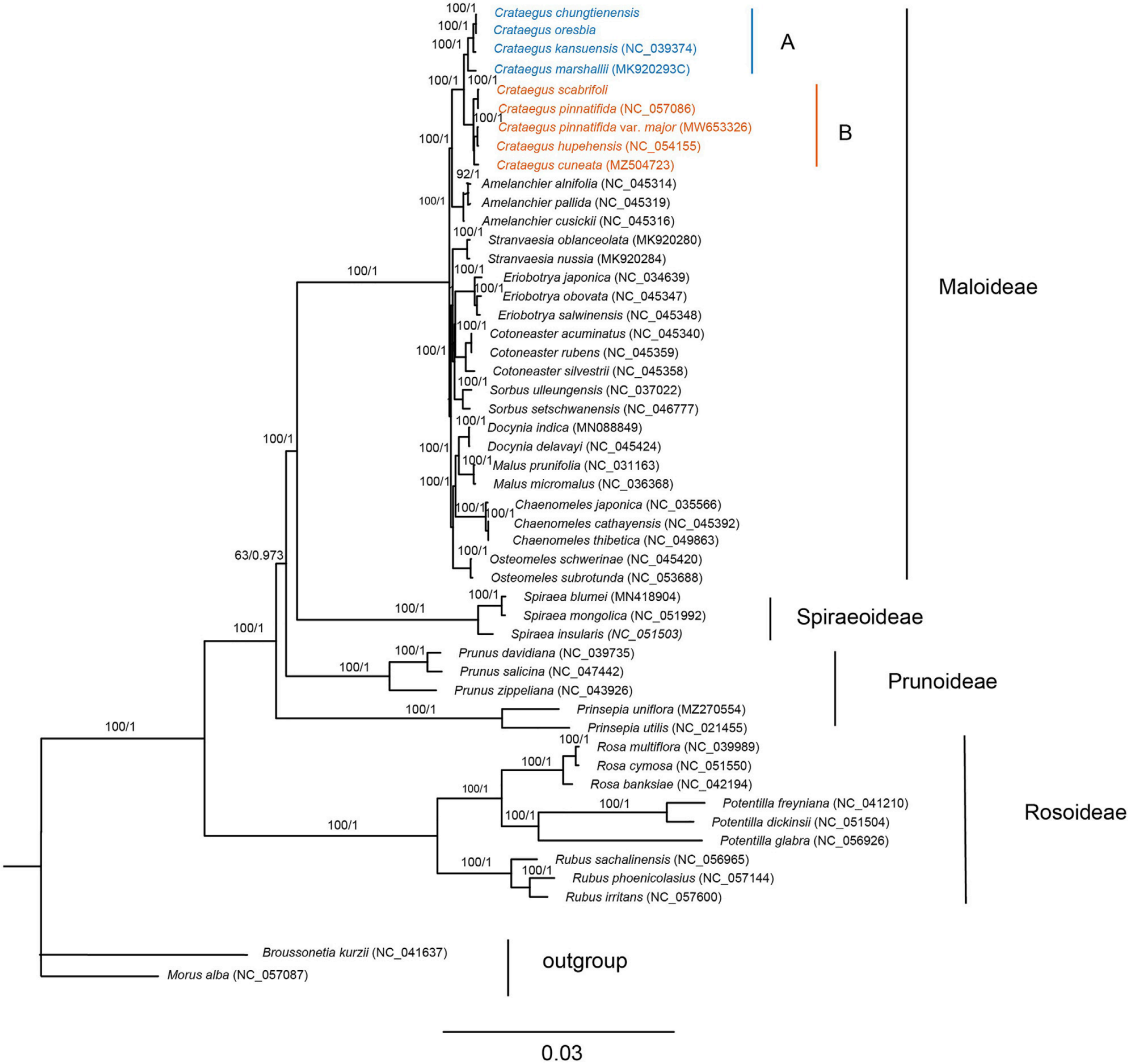
Phylogenetic tree of *Crataegus* within the Rosaceae. The entire genome data set was analyzed using maximum likelihood (ML) and Bayesian information (BI). Different colors represent different clades **(A,B)**.

**FIGURE 7:**
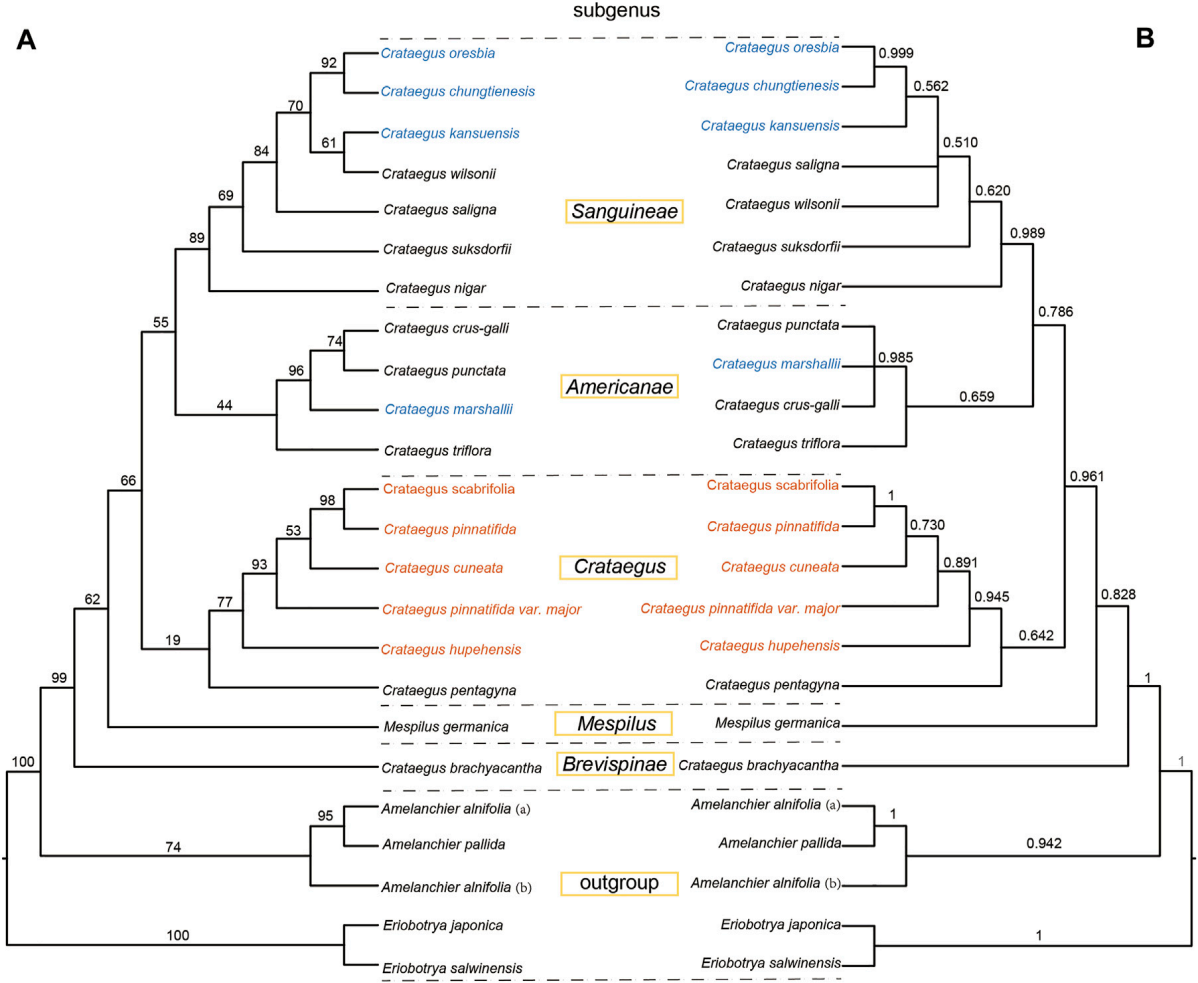
Phylogenetic tree of *Crataegus* based on the sequences of five intergenic regions. The different colors represent the different evolutionary branches clades (clade A and clade B) in the phylogenetic tree constructed from the complete chloroplast genome. **(A)** Phylogenetic tree constructed by maximum likelihood (ML). **(B)** Phylogenetic tree constructed by maximum Bayesian method (BI).

Using a total of 50 chloroplast genomes, including 48 species of Rosaceae with two species of Moraceae as outgroups, the phylogenetic relationships of Rosaceae plants were studied using ML and BI phylogenetic analysis. The topologies of the two datasets (ML and BI) were basically the same, with high support for each branch. Two subfamilies (Amygdaloideae and Rosoideae) and seven tribes (Maleae, Spiraeeae, Amygdaleae, Exochordeae, Roseae, Potentilleae, and Rubeae) were strongly supported as monophyletic groups. It was notable that species in *Crataegus* were divided into two clades, clade A included *C. chungtienensis*, *C. oresbia*, *C. kansuensis* and *C. marshallii*, and clade B included *C. scabrifoli*, *C. pinnatifida*, *C. pinnatifida* var. *major*, *C. hupehensis* and *C. cuneata* (Figure 6). That was consistent with previous results (Zhang et al., 2017; Hu et al., 2021a; Wu et al., 2021). The phylogenetic trees of the intergenic regions resolved 19 hawthorn species into five clades. *C. pinnatifida*, *C. pinnatifida* var. *major*, *C. hupehensis*, *C. cuneata* and *C. scabrifolia* clustered with species from C. subg. *Crataegus*. *C. kansuensis*, *C. oresbia* and *C. kansuensis* clustered with species from *C*. subg. *Sanguineae*. *C. marshallii* clustered with species from *C*. subg. *Americanae*. Two species (*C. germanica* and *C. brachyacantha*) from *C*. subg. *Mespilus* and *C*. subg. *Brevispinae*, respectively (Figure 7).

## 4 Discussion

### 4.1 Chloroplast Genome Structure

The newly sequenced three chloroplast genomes of *Crataegus* are similar in composition; the structures are the same as with other angiosperms (Liu et al., 2018; Gao et al., 2019; Zhang X-F et al., 2021). In addition, the chloroplast genomes of *C. pinnatifida* var. *major*, *C. cuneata* and *C. pinnatifida* lacked the *infA* and this is the most common gene loss event in the chloroplast genome of angiosperms (Liu et al., 2017; Li et al., 2021). Studies have shown that *infA* is an unstable chloroplast gene that is lost from the chloroplast genome and transferred several times to the nucleus (Millen et al., 2001).

Correlation analysis of SNPs, indels and repeats showed weak correlations (*p* < 0.001) between indels and SNPs in four species of Rosaceae (*E. salwinensis*, *M. micromalus*, *S. mongolica*, R*. phoenicolasius*). In the correlation analysis of indels with SSRs and repeats, *C. scabrifoli*, *E. salwinensis* and *M. micromalus* showed moderate correlation, while the other two species showed no correlation (Table 3). SNPs and repeats showed no correlation. However, in the co-occurrence analysis of repeats with indels and SNPs, a total of 98 SSRs were detected, of which 75 (76.53%) co-occurred with indels and 70 (71.42%) co-occurred with SNPs (Supplementary Table S6). In addition, Among the 49 oligonucleotide repeats detected, 38 (77.55%) co-occurred with indels and 40 (81.63%) co-occurred with SNPs (Supplementary Table S7). Previous studies have also reported high co-occurrence of repeats with indels and SNPs, and suggested a role for repeats in SNPs and indel mutations (Ibrar et al., 2012; Abdullah et al., 2020; Liu et al., 2020). We believe that the effect of repeats on genetic variation cannot be ignored. Based on the high co-occurrence between oligonucleotide repeats and indels, SNPs, we support the ideal of using oligonucleotide repeats as a proxy for finding mutational hotspots (Abdullah et al., 2020).

**TABLE 3:** Correlation values of indels with SNPs, indels with repeats and SNPs with repeats.

	Indels and SNPs		Indels and SSRs		Indels and Oligonucleotide repeats		Indels and Repeats		SNPs and SSRs		SNPs and Oligonucleotide repeats		SNPs and Repeats	
	Rho	*p*-value	Rho	*p*-value	Rho	*p*-value	Rho	*p*-value	Rho	*p*-value	Rho	*p*-value	Rho	*p*-value
*C. scabrifoli*	0.161	<0.001	0.332	<0.001	0.093	0.002	0.343	<0.001	0.068	0.026	0.007	0.819	0.065	0.034
*E. salwinensis*	0.211	<0.001	0.335	<0.001	0.053	0.081	0.323	<0.001	0.088	0.004	−0.015	0.613	0.07	0.023
*M, micromalus*	0.202	<0.001	0.358	<0.001	0.071	0.02	0.345	<0.001	0.057	0.060	−0.023	0.455	0.037	0.228
*S, mongolica*	0.256	<0.001	0.188	<0.001	0.003	0.916	0.156	<0.001	−0.020	0.470	−0.117	<0.001	−0.08	0.009
*R.phoenicolasius*	0.286	<0.001	0.047	0.125	−0.024	0.431	0.032	0.290	−0.059	0.129	−0.114	<0.001	−0.095	0.002

### 4.2 Identification of Highly Variable Regions

Highly variable regions containing rich informative loci can serve as DNA barcodes to construct phylogenetic trees, identify closely related species and accelerate the discovery of species not yet found in nature (Kress and Erickson, 2008; Dong et al., 2012; Li et al., 2015). In this study, six highly variable regions were identified, including *rps16-trnQ-*UUG, *ndhF-rpl32*, *rps16-psbK*, *trnR-*UCU*-atpA*, *rpl32-trnL*-UAG, and *ndhA*. Some intergenic regions (*trnG-trnS*, *rpl20-rps12*, *trnL-trnF*, *psbA-trnH*, *trnC-ycf6*, and *trnH-rpl2*) and gene regions (*rps16* intron, *rpl16* intron, *accD*, *rpoC1*, *rbcL*, and *ndhF*) have been used in phylogenetic studies of the Rosaceae (Lo et al., 2009; Lo and Donoghue, 2012; Zarrei et al., 2015). In addition, studies have shown that the resolution of plant universal DNA barcodes (*rbcL*, *matK*, *psbA-trnH*, and ITS2) for taxa of the genus *Crataegus* was poor. In contrast, concatenated sequences of three plastid barcode loci and 11 other plastid loci provided better resolution (Zarrei et al., 2015). Therefore, these highly variable regions provide abundant information about molecular marker development for plant identification and phylogenetic relationships in *Crataegus*.

### 4.3 Phylogenomic Validation

Because complete chloroplast genome sequences are not yet available for some species, we used intergenic regions to construct phylogenetic trees and analyze the relationship between species within the *Crataegus* subgenera. Some segments extracted from the complete chloroplast genome (such as *psbA-trnH*, *rps16*, etc.) differ significantly from the sequences obtained by NCBI. Therefore, we used only five intergenic regions that could be accurately extracted to construct the phylogenetic tree. The results of this phylogenetic analysis also provided us with some valuable information. *C. pinnatifida*, *C. pinnatifida* var. *major*, *C. hupehensis*, *C. cuneata* and *C. scabrifolia* clustered with species from *C.* subg. *Crataegus*, while *C. kansuensis*, *C. oresbia* and *C. kansuensis* clustered with species from *C.* subg. *Sanguineae* (Figure 7). This result is consistent with the phylogenetic tree of the complete chloroplast genome, and the eight species (*C. marshalli* is a species from North America) native to China were divided into two clades (clade A and clade B). Previous classifications based on morphology placed *C. pinnatifida* (*C.* sect. *Pinnatifidae* Zabel ex C.K.Schneid.), *C. hupehensis* (*C.* sect. *Hupehenses* J.B.Phipps), *C. cuneata* (*C.* sect. *Cuneatae* Rehder ex C.K. Schneid), and *C. scabrifolia* (*C.* sect. *Henryanae* (Sarg.) J.B.Phipps) in *C.* subg. *Crataegus* (Ufimov and Dickinson, 2020). However, it is noteworthy that the leaf morphology of *C. scabrifolia* (unlobed leaves) is different from other species (lobed leaves) in clade A (Gu and Spongberg, 2003). Hybridization of different species is one of the reasons for the diversity of morphology in *Crataegus* (Lo et al., 2009; Zarrei et al., 2015). Previous studies have documented a hybrid (*Crataegus* × *cogswellii* K.I. Chr. & T.A.) with a leaf shape between unlobed [*C. suksdorfii* (Sarg.) Kruschke] and markedly lobed (*Crataegus monogyna* Jacq.) (Christensen et al., 2014). In addition, studies have shown that hawthorn species native to China may have two evolutionary pathways, one is that *C. scabrifolia* differentiated or hybridized to produce new species during its northward migration (Du et al., 2019). Although there are significant differences in leaf morphology between species in clade A, we agree with the view that they have the same origin, and support the four sections (*C.* sect. *Pinnatifidae* Zabel ex C.K.Schneid., *C.* sect. *Hupehenses* J.B.Phipps, *C.* sect. *Cuneatae* Rehder ex C.K. Schneid and *C.* sect. *Henryanae* (Sarg.) J.B.Phipps) in *C*. subg. *Crataegus*.

Another evolutionary pathway of hawthorns native to China is closely related to the North American species (Du et al., 2019). Our results also confirm this view, *C. scabrifolia*, *C. chungtienensis*, and *C. oresbia* are all from southwestern China; phylogenetic tree results indicate that *C. scabrifolia* is distant from the other two species. *C. scabrifolia* is also morphologically significantly different from *C. chungtienensis* and *C. oresbia* (Supplementary Table S8). In addition, research suggests that the ancient trans-Beringian migrations made the species of East Asia and western North America closely related (Phipps, 1983; Lo et al., 2009). Our results are consistent with this study (Lo et al., 2009), *C. kansuensis*, *C. oresbia*, *C. kansuensis* and *C. wilsonii* from East Asia are more closely related to *C. saligna* and *C. suksdorfii* from western North America, while they are distant from *C. marshallii*, *C. punctata*, *C. crus-galli* and *C. triflora* from Eastern America. Therefore, we support the morphology-based classification results that place *C. kansuensis*, *C. oresbia*, *C. kansuensis* in *C.* subg. *Sanguineae.* In addition, *C. marshallii* is considered a paleohybrid, and its maternal parent may be a species closely related to *C. Mexicana* (*C.* subg. *American*) (Lo et al., 2009). Therefore, C. marshallii clustered with species of the *C.* subg. *American* after adding species from this subgenus. It is worth noting that most species in clade B are used as cultivated varieties and medicinal materials, and it is generally believed that hawthorn in clade B may have more value. Most of the current studies have focused on species associated with clade B, while fewer studies have been conducted on species associated with clade A (Cui et al., 2022; Liu et al., 2017; Lou et al., 2022). However, it has been found that the concentration of vitexin derivatives in the leaves of European hawthorn was significantly higher than that of North American hawthorn, while the rutin content of North American hawthorn was significantly higher than European hawthorn (Lund et al., 2020). The active constituents of related species generally have similar chemical constituents and functions. Therefore, the research on clade A hawthorn species is of great significance.

According to the results of the phylogenetic tree, *C. chungtienensis* and *C. oresbia* are closely related, and the two species are also very similar in morphology. Both *C. chungtienensis* and *C. oresbia* have the following morphological characters (Supplementary Table S8): they are all shrubs, about 6 m tall; the leaves are broadly ovate and usually with 3-5 pairs of shallow lobes, leaf blade abaxially sparsely pubescent; corymb; bracts caducous; pomes are red and 0.6 cm in diameter; pyrenes with concave scars on both inner sides. The main difference between the two species is the white tomentose visible on the peduncle of *C. oresbia* (Gu and Spongberg, 2003). In addition, the geographical distribution of these two species is highly overlapping, mainly distributed in the alpine regions of southwest China (such as Zhongdian and Weixi), with an altitude of about 2,500–3,300 m (Gu and Spongberg, 2003). Based on the results of molecular evidence, morphological characteristics, and geographical distribution, we speculate that *C. chungtienensis* and *C. oresbia* may be the same species.

## 5 Conclusion

In this study, we sequenced the complete chloroplast genomes of three species of *Crataegus*, and comparatively analyzed them with other species in Rosaceae. The chloroplast genome features of *Crataegus* were similar to the other angiosperms, including gene size, genome structure, gene number and gene order. In addition, we found some highly divergent regions, which provided valuable information for the identification and phylogenetic relationships of *Crataegus*. Phylogenetic studies based on the complete chloroplast genome revealed that *Crataegus* can be divided into 2 clades; there are some key differences between the two clades in morphological characteristics and application value. We constructed a phylogenetic tree using intergenic regions and discussed the subgenus classification of eight hawthorn species native to China. We speculate that *C. chungtienensis* and *C. oresbia* are the same species. The major limitations of this study were the small sample sizes. In future work, more taxa could be used and more powerful tools including transcriptome, and whole-genomes could be included to shed light on the evolutionary history of *Crataegus*. Nevertheless, this study provides an important step in further understanding the phylogeny of the genus and the taxonomic relationships among these species.

## Data Availability

The datasets presented in this study can be found in online repositories. The names of the repository/repositories and accession number(s) can be found below: GenBank, accessions numbers: ON032469, ON032470 and ON032470.

## Supplementary Material

Supplementary Figure S1 Photos of Crataegus oresbia and Crataegus kansuensis. **(A,B,C)** Crataegus oresbia. **(D,E,F)** Crataegus kansuensis.

Supplementary Figure S2 Analysis of amino acid abundance among the 9 species of *Crataegus* chloroplast genomes.

Supplementary Figure S3 Analysis of simple repeat sequences (SSR) and long repeats among the nine species of *Crataegus* chloroplast genomes. **(A)** Number of different SSR types detected in 9 *Crataegus* chloroplast genomes. **(B)** Frequency rates of identified SSR motifs in different repeat class types. **(C)** Number of four types of long repeats. **(D)** Number of different lengths of long repeats.

## References

[B1] Abdullah, Mehmood F. Shahzadi I. Ali Z. Islam M. Naeem M. (2020). Correlations Among Oligonucleotide Repeats, Nucleotide Substitutions, and Insertion-Deletion Mutations in Chloroplast Genomes of Plant Family Malvaceae. J. Syst. Evol. 59, 388–402. 10.1111/jse.12585

[B2] Ahmed I. Biggs P. J. Matthews P. J. Collins L. J. Hendy M. D. Lockhart P. J. (2012). Mutational Dynamics of Aroid Chloroplast Genomes. Genome Biol. Evol. 4, 1316–1323. 10.1093/gbe/evs110 23204304 PMC3542561

[B3] Amiryousefi A. Hyvönen J. Poczai P. (2018). IRscope: An Online Program to Visualize the Junction Sites of Chloroplast Genomes. Bioinformatics 34, 3030–3031. 10.1093/bioinformatics/bty220 29659705

[B4] Beier S. Thiel T. Münch T. Scholz U. Mascher M. (2017). MISA-Web: a Web Server for Microsatellite Prediction. Bioinformatics 33, 2583–2585. 10.1093/bioinformatics/btx198 28398459 PMC5870701

[B5] Bennetzen J. L. Ma J. Devos K. (2005). Mechanisms of Recent Genome Size Variation in Flowering Plants. Ann. Bot. 95, 127–132. 10.1093/aob/mci008 15596462 PMC4246713

[B6] Biju V. C. P.R. S. Vijayan S. Rajan V. S. Sasi A. Janardhanan A. (2019). The Complete Chloroplast Genome of Trichopus Zeylanicus , and Phylogenetic Analysis with Dioscoreales. Plant Genome 12, 190032. 10.3835/plantgenome2019.04.0032 PMC12810117 33016590

[B7] Bremer B. Bremer K. Heidari N. Erixon P. Olmstead R. G. Anderberg A. A. (2002). Phylogenetics of Asterids Based on 3 Coding and 3 Non-coding Chloroplast DNA Markers and the Utility of Non-coding DNA at Higher Taxonomic Levels. Mol. Phylogenetics Evol. 24, 274–301. 10.1016/S1055-7903(02)00240-3 12144762

[B8] Christensen K. I. Zarrei M. Kuzmina M. Talent N. Lin C. Dickinson T. (2014). Crataegus ×ninae-Celottiae and C. ×cogswellii (Rosaceae, Maleae), Two Spontaneously Formed Intersectional Nothospecies. Phytokeys 36, 1–26. 10.3897/phytokeys.36.6784 PMC4023339 24843290

[B65] Cui Y. Du K. Hou S. Yang R. Qi L. Li J. (2022). A Comprehensive Strategy Integrating Metabolomics With Multiple Chemometric for Discovery of Function Related Active Markers for Assessment of Foodstuffs: A Case of Hawthorn (Crataegus cuneata) Fruits. Food Chem. 383, 132464. 10.1016/j.foodchem.2022.132464 35189445

[B9] Dong P. Pan L. Zhang X. Zhang W. Wang X. Jiang M. (2017). Hawthorn ( Crataegus Pinnatifida Bunge) Leave Flavonoids Attenuate Atherosclerosis Development in apoE Knock-Out Mice. J. Ethnopharmacol. 198, 479–488. 10.1016/j.jep.2017.01.040 28119096

[B10] Dong W. Liu J. Yu J. Wang L. Zhou S. (2012). Highly Variable Chloroplast Markers for Evaluating Plant Phylogeny at Low Taxonomic Levels and for DNA Barcoding. PLoS ONE 7, e35071. 10.1371/journal.pone.0035071 22511980 PMC3325284

[B67] Du X. Zhang X. Bu H. Zhang T. Lao Y. Dong Y. (2019). Molecular Analysis of Evolution and Origins of Cultivated Hawthorn (spp.) and Related Species in China. Front. Plant Sci. 10, 443. 10.3389/fpls.2019.00443 31024604 PMC6465762

[B11] Evans R. C. Campbell C. S. (2002). The Origin of the Apple Subfamily (Maloideae; Rosaceae) Is Clarified by DNA Sequence Data from Duplicated GBSSI Genes. Am. J. Bot. 89, 1478–1484. 10.3732/ajb.89.9.1478 21665749

[B12] Gao B. Yuan L. Tang T. Hou J. Pan K. Wei N. (2019). The complete chloroplast genome sequence of *Alpinia oxyphylla* Miq. and comparison analysis within the Zingiberaceae family. PLoS ONE 14, e0218817. 10.1371/journal.pone.0218817 31233551 PMC6590956

[B13] Gascuel O. (1997). BIONJ: An improved version of the NJ algorithm based on a simple model of sequence data. Mol. Biol. Evol. 14, 685–695. 10.1093/oxfordjournals.molbev.a025808 9254330

[B14] Greiner S. Lehwark P. Bock R. (2019). OrganellarGenomeDRAW (OGDRAW) version 1.3.1: expanded toolkit for the graphical visualization of organellar genomes. Nucleic Acids Res. 47, W59–W64. 10.1093/nar/gkz238 30949694 PMC6602502

[B15] Gu C. Spongberg S. A. (2003). “ *Crataegus* Linnaeus,” in *Flora of China*. Vol 9 (Pittosporaceae Through Connaraceae). Editors Wu Z. Y. Raven P. H. Hong D. Y. (Beijing: Science Press), 111–117.

[B16] He S.-L. Xie J. Yang Y. Tian Y. (2020). Chloroplast genome for Crataegus pinnatifida (Rosaceae) and phylogenetic analyses with its coordinal species. Mitochondrial DNA Part B 5, 2097–2098. 10.1080/23802359.2019.1667273 33366930 PMC7510473

[B17] Hollingsworth P. M. Hollingsworth P. M. Forrest L. L. Spouge J. L. Hajibabaei M. Ratnasingham S. (2009). A DNA barcode for land plants. Proc. Natl. Acad. Sci. U.S.A. 106, 12794–12797. 10.1073/pnas.0905845106 19666622 PMC2722355

[B18] Hu G. Wang Y. Wang Y. Zheng S. Dong W. Dong N. (2021a). New insight into the phylogeny and taxonomy of cultivated and related species of *Crataegus* in China, based on complete chloroplast genome sequencing. Horticulturae 7, 301. 10.3390/horticulturae7090301

[B19] Hu G. Zheng S. Pan Q. Dong N. (2021b). The complete chloroplast genome of *Crataegus hupehensis* Sarg. (Rosaceae), a medicinal and edible plant in China. Mitochondrial DNA Part B 6, 315–317. 10.1080/23802359.2020.1866464 33659661 PMC7872584

[B20] Hu Y. Ma Z. Dang M. Feng X. Sun Y. Yuan X. (2017). The complete plastid genome of the endangered species midget crabapple (*Malus micromalus*). Conserv. Genet. Resour. 10, 531–533. 10.1007/s12686-017-0867-1

[B21] Jurikova T. Sochor J. Rop O. Mlcek J. Balla S. Szekeres L. (2012). Polyphenolic profile and biological activity of Chinese hawthorn (*Crataegus pinnatifida* BUNGE) fruits. Molecules 17, 14490–14509. 10.3390/molecules171214490 23222867 PMC6268084

[B22] Katayama H. Uematsu C. (2005). Structural analysis of chloroplast DNA in *Prunus* (Rosaceae): evolution, genetic diversity and unequal mutations. Theor. Appl. Genet. 111, 1430–1439. 10.1007/s00122-005-0075-3 16142464

[B23] Khakhlova O. Bock R. (2006). Elimination of deleterious mutations in plastid genomes by gene conversion. Plant J. 46, 85–94. 10.1111/j.1365-313X.2006.02673.x 16553897

[B24] Kress W. J. Erickson D. L. (2008). DNA barcodes: Genes, genomics, and bioinformatics. Proc. Natl. Acad. Sci. U.S.A. 105, 2761–2762. 10.1073/pnas.0800476105 18287050 PMC2268532

[B25] Kurtz S. Choudhuri J. Ohlebusch E. Schleiermacher C. Stoye J. Giegerich R. (2001). REPuter: the manifold applications of repeat analysis on a genomic scale. Nucleic Acids Res. 29, 4633–4642. 10.1093/nar/29.22.4633 11713313 PMC92531

[B26] Li F. Xie X. Huang R. Tian E. Li C. Chao Z. (2021). Chloroplast genome sequencing based on genome skimming for identification of *Eriobotryae Folium* . BMC Biotechnol. 21, 69. 10.1186/s12896-021-00728-0 34895202 PMC8666020

[B27] Li X. Tan W. Sun J. Du J. Zheng C. Tian X. (2019). Comparison of four complete chloroplast genomes of medicinal and ornamental *Meconopsis* species: genome organization and species discrimination. Sci. Rep. 9, 10567. 10.1038/s41598-019-47008-8 31332227 PMC6646306

[B28] Li X. Yang Y. Henry R. J. Rossetto M. Wang Y. Chen S. (2015). Plant DNA barcoding: from gene to genome. Biol. Rev. 90, 157–166. 10.1111/brv.12104 24666563

[B29] Librado P. Rozas J. (2009). DnaSP v5: a software for comprehensive analysis of DNA polymorphism data. Bioinformatics 25, 1451–1452. 10.1093/bioinformatics/btp187 19346325

[B30] Liston A. Weitemier K. A. Letelier L. Podani J. Zong Y. Liu L. (2021). Phylogeny of Crataegus (Rosaceae) based on 257 nuclear loci and chloroplast genomes: evaluating the impact of hybridization. PeerJ 9, e12418. 10.7717/peerj.12418 34754629 PMC8555502

[B31] Liu B.-B. Hong D. Y. Zhou S. L. Xu C. Dong W. P. Johnson G. (2019a). Phylogenomic analyses of the Photinia complex support the recognition of a new genus *Phippsiomeles* and the resurrection of a redefined Stranvaesia in Maleae (Rosaceae). Jnl Sytematics Evol. 57, 678–694. 10.1111/jse.12542

[B32] Liu B.-B. Liu G.-N. Hong D.-Y. Wen J. (2019b). Eriobotrya Belongs to Rhaphiolepis (Maleae, Rosaceae): Evidence From Chloroplast Genome and Nuclear Ribosomal DNA Data. Front. Plant Sci. 10, 1731. 10.3389/fpls.2019.01731 32117331 PMC7019104

[B33] Liu H.-Y. Yu Y. Deng Y.-Q. Li J. Huang Z.-X. Zhou S.-D. (2018). The Chloroplast Genome of *Lilium henrici*: Genome Structure and Comparative Analysis. Molecules 23, 1276. 10.3390/molecules23061276 PMC6100032 29861452

[B34] Liu L.-X. Li R. Worth J. R. P. Li X. Li P. Cameron K. M. (2017). The Complete Chloroplast Genome of Chinese Bayberry (*Morella rubra*, Myricaceae): Implications for Understanding the Evolution of Fagales. Front. Plant Sci. 8, 968. 10.3389/fpls.2017.00968 28713393 PMC5492642

[B35] Liu Q. Li X. Li M. Xu W. Schwarzacher T. Heslop-Harrison J. S. (2020). Comparative chloroplast genome analyses of *Avena*: Insights into evolutionary dynamics and phylogeny. BMC Plant Biol. 20, 406. 10.1186/s12870-020-02621-y 32878602 PMC7466839

[B36] Lo E. Y. Y. Donoghue M. J. (2009). Expanded phylogenetic and dating analyses of the apples and their relatives (Pyreae, Rosaceae). Mol. Phylogenetics Evol. 52, 230–243. 10.1016/j.ympev.2011.10.005 22293154

[B37] Lo E. Y. Y. Donoghue M. J. (2012). Expanded phylogenetic and dating analyses of the apples and their relatives (Pyreae, Rosaceae). Mol. Phylogenetics Evol. 63, 230–243. 10.1016/j.ympev.2011.10.005 22293154

[B38] Lo E. Y. Y. StefanovićChristensen S. Christensen K. I. Dickinson T. A. (2009). Evidence for genetic association between East Asian and western North American *Crataegus* L. (Rosaceae) and rapid divergence of the eastern North American lineages based on multiple DNA sequences. Mol. Phylogenetics Evol. 51, 157–168. 10.1016/j.ympev.2009.01.018 19405185

[B39] Lohse M. Drechsel O. Bock R. (2007). OrganellarGenomeDRAW (OGDRAW): A tool for the easy generation of high-quality custom graphical maps of plastid and mitochondrial genomes. Curr. Genet. 52, 267–274. 10.1007/s00294-007-0161-y 17957369

[B64] Lou X. Jin Y. Tian H. Yu H. Chen C. Hanna M. (2022). High-pressure and Thermal Processing of Cloudy Hawthorn Berry (Crataegus pinnatifida) Juice: Impact on Microbial Shelf-Life, Enzyme Activity and Quality-Related Atributes. Food Chem. 372, 131313. 10.1016/j.foodchem.2021.131313 34655827

[B63] Lund J. Brown P. Shipley P. (2020). Quantification of North American and European Crataegus Flavonoids by Nuclear Magnetic Resonance Spectrometry. Fitoterapia. 143, 104537. 10.1016/j.fitote.2020.104537 32145312

[B40] Luo C. Huang W. Sun H. Yer H. Li X. Li Y. (2021). Comparative chloroplast genome analysis of Impatiens species (Balsaminaceae) in the karst area of China: insights into genome evolution and phylogenomic implications. BMC Genomics 22, 571. 10.1186/s12864-021-07807-8 34303345 PMC8310579

[B41] Ma Y. Guo Y. Zhu Y. Liu Q. Gao L. Yang W. (2021). The complete chloroplast genome of *Spiraea mongolica* Maxim. Mitochondrial DNA Part B 6, 1614–1616. 10.1080/23802359.2021.1926351 34027071 PMC8118401

[B42] Margulis L. (1976). Genetic and evolutionary consequences of symbiosis. Exp. Parasitol. 39, 277–349. 10.1016/0014-4894(76)90127-2 816668

[B43] Millen R. S. Olmstead R. G. Adams K. L. Palmer J. D. Lao N. T. Heggie L. (2001). Many Parallel Losses of *infA* from chloroplast DNA during angiosperm evolution with multiple independent transfers to the nucleus. Plant Cell 13, 645–658. 10.1105/tpc.13.3.645 11251102 PMC135507

[B44] Nguyen H. Q. Nguyen T. N. L. Doan T. N. Nguyen T. T. N. Phạm M. H. Le T. L. (2021). Complete chloroplast genome of novel *Adrinandra megaphylla* Hu species: molecular structure, comparative and phylogenetic analysis. Sci. Rep. 11, 11731. 10.1038/s41598-021-91071-z 34083611 PMC8175739

[B45] Palmer J. D. (1991). Plastid chromosomes: structure and evolution. Mol. Biol. Plastids 1997, 5–53. 10.1016/B978-0-12-715007-9.50009-8

[B46] Phipps J. B. (1983). Biogeographic, Taxonomic, and Cladistic Relationships Between East Asiatic and North American *Crataegus* . Ann. Mo. Botanical Gard. 70, 667–700. 10.2307/2398984

[B47] Phipps J. B. Robertson K. R. Smith P. G. Rohrer J. R. (1990). A checklist of the subfamily Maloideae (Rosaceae). Can. J. Bot. 68, 2209–2269. 10.1139/b90-288

[B68] Seemann T. (2015). Snippy: Fast Bacterial Variant Calling From NGS Reads. Availabe at: https://github.com/tseemann/snippy .

[B48] Shaw J. Lickey E. B. Beck J. T. Farmer S. B. Liu W. Miller J. (2005). The tortoise and the hare II: relative utility of 21 noncoding chloroplast DNA sequences for phylogenetic analysis. Am. J. Bot. 92, 142–166. 10.3732/ajb.92.1.142 21652394

[B49] Shinozaki K. Ohme M. Tanaka M. Wakasugi T. Hayashida N. Matsubayashi T. (1986). The complete nucleotide sequence of the tobacco chloroplast genome: its gene organization and expression. EMBO J. 5, 2043–2049. 10.1002/j.1460-2075.1986.tb04464.x 16453699 PMC1167080

[B50] Ufimov R. A. Dickinson T. A. (2020). Infrageneric nomenclature adjustments in *Crataegus* L. (Maleae, Rosaceae). Phytologia 102, 177–199. 10.7934/p3190

[B51] Wolfe K. H. Li W. H. Sharp P. M. (1987). Rates of nucleotide substitution vary greatly among plant mitochondrial, chloroplast, and nuclear DNAs. Proc. Natl. Acad. Sci. U.S.A. 84, 9054–9058. 10.1073/pnas.84.24.9054 3480529 PMC299690

[B52] Wu L. Cui Y. Wang Q. Xu Z. Wang Y. Lin Y. (2021). Identification and phylogenetic analysis of five *Crataegus* species (Rosaceae) based on complete chloroplast genomes. Planta 254, 14. 10.1007/s00425-021-03667-4 34180013

[B53] Wyman S. K. Jansen R. K. Boore J. L. (2004). Automatic annotation of organellar genomes with DOGMA. Bioinformatics 20, 3252–3255. 10.1093/bioinformatics/bth352 15180927

[B54] Yu J. Wu X. Liu C. Newmaster S. Ragupathy S. Kress W. J. (2021). Progress in the use of DNA barcodes in the identification and classification of medicinal plants. Ecotoxicol. Environ. Saf. 208, 111691. 10.1016/j.ecoenv.2020.111691 33396023

[B55] Zarrei M. Talent N. Kuzmina M. Lee J. Lund J. Shipley P. R. (2015). DNA barcodes from four loci provide poor resolution of taxonomic groups in the genus *Crataegus* . AoB Plants 7, plv045. 10.1093/aobpla/plv045 25926325 PMC4480070

[B56] Zhang D. Gao F. Jakovlić I. Zou H. Zhang J. Li W. X. (2020). PhyloSuite: An integrated and scalable desktop platform for streamlined molecular sequence data management and evolutionary phylogenetics studies. Mol. Ecol. Resour. 20, 348–355. 10.1111/1755-0998.13096 31599058

[B57] Zhang G. Liu Y. Hai P. (2021). The complete chloroplast genome of Tibetan medicinal plant Rubus phoenicolasius Maxim. Mitochondrial DNA Part B 6, 886–887. 10.1080/23802359.2021.1886013 33796668 PMC7971196

[B58] Zhang S. D. Jin J. J. Chen S. Y. Chase M. W. Soltis D. E. Li H. T. (2017). Diversification of Rosaceae since the Late Cretaceous based on plastid phylogenomics. New Phytol. 214, 1355–1367. 10.1111/nph.14461 28186635

[B59] Zhang X.-F. Landis J. B. Wang H.-X. Zhu Z.-X. Wang H.-F. (2021). Comparative analysis of chloroplast genome structure and molecular dating in Myrtales. BMC Plant Biol. 21, 219. 10.1186/s12870-021-02985-9 33992095 PMC8122561

[B60] Zhang X. Wang Y. Wang M. Yuan Q. Huang L. (2020). The complete chloroplast genome of the *Crataegus kansuensis* (Rosaceae): characterization and phylogeny. Mitochondrial DNA Part B 5, 2920–2921. 10.1080/23802359.2020.1792368 33458002 PMC7782147

[B61] Zhao J. Xu Y. Xi L. Yang J. Chen H. Zhang J. (2018). Characterization of the chloroplast genome sequence of *Acer miaotaiense*: comparative and phylogenetic analyses. Molecules 23, 1740. 10.3390/molecules23071740 PMC6099587 30018192

[B62] Zhu R.-G. Sun Y.-D. Li T.-P. Chen G. Peng X. Duan W.-B. (2015). Comparative effects of hawthorn (*Crataegus pinnatifida* Bunge) pectin and pectin hydrolyzates on the cholesterol homeostasis of hamsters fed high-cholesterol diets. Chemico-Biological Interact. 238, 42–47. 10.1016/j.cbi.2015.06.006 26070415

