# Supplementary figures and images for: Comparative Genomic and Phylogenetic Analysis of Chloroplast Genomes of Hawthorn (Crataegus spp.) in Southwest China
Source: Front Genet. 2022 Jul 4;13:900357. doi: 10.3389/fgene.2022.900357 (PMC9289535; doi:10.3389/fgene.2022.900357)

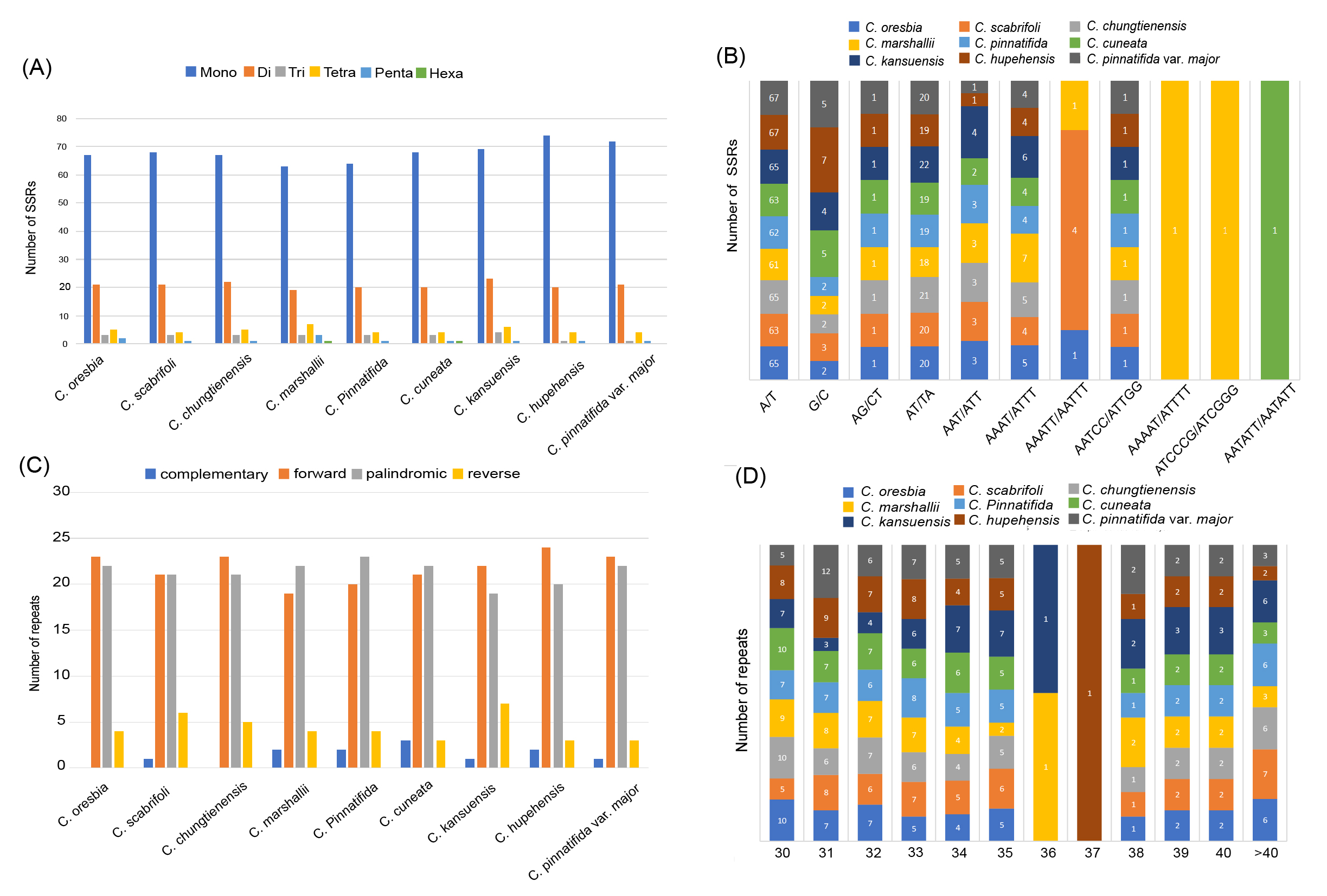

Supplement: Supplementary file 2 [file Image3.JPEG]

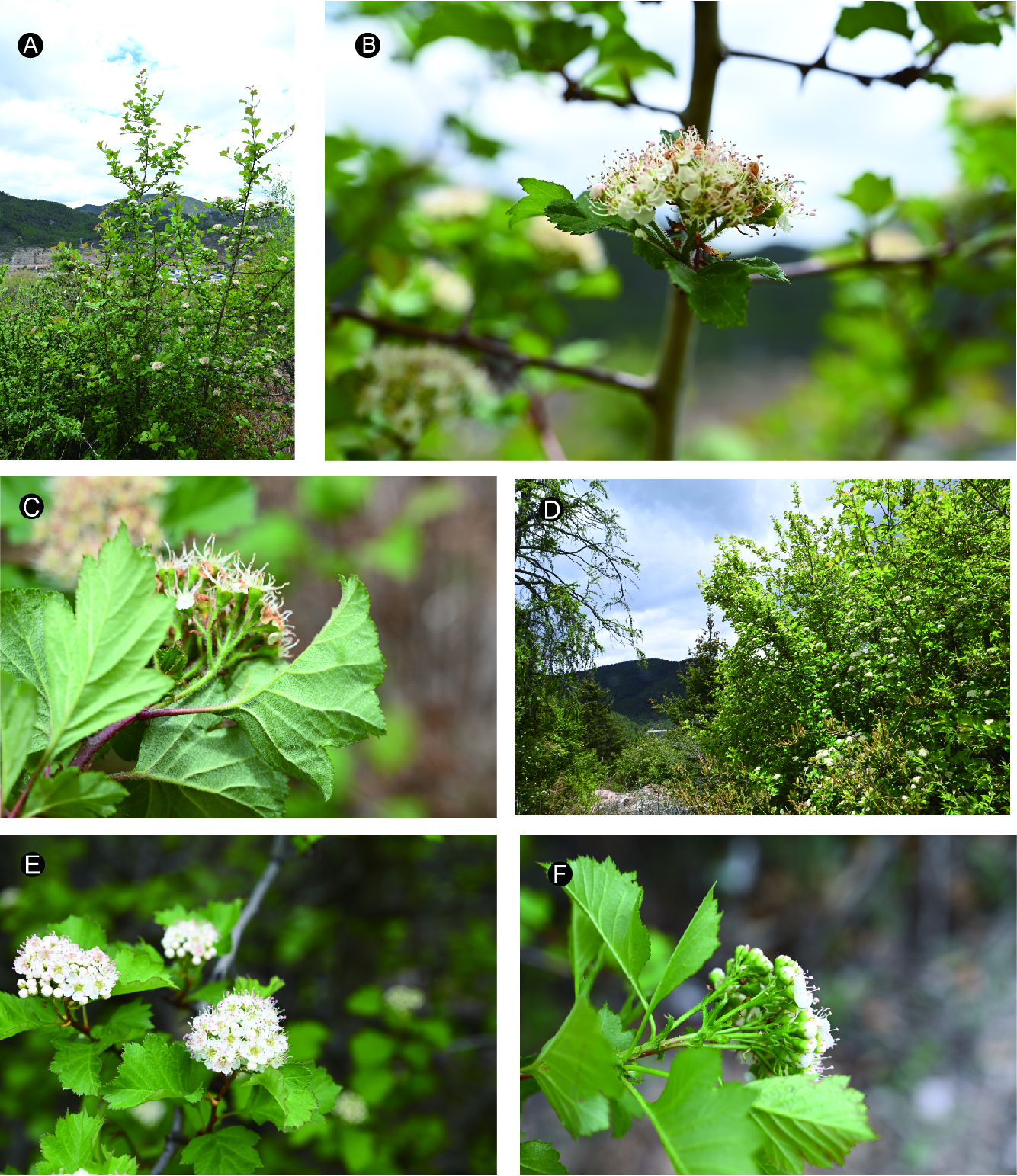

Supplement: Supplementary file 5 [file Image1.JPEG]

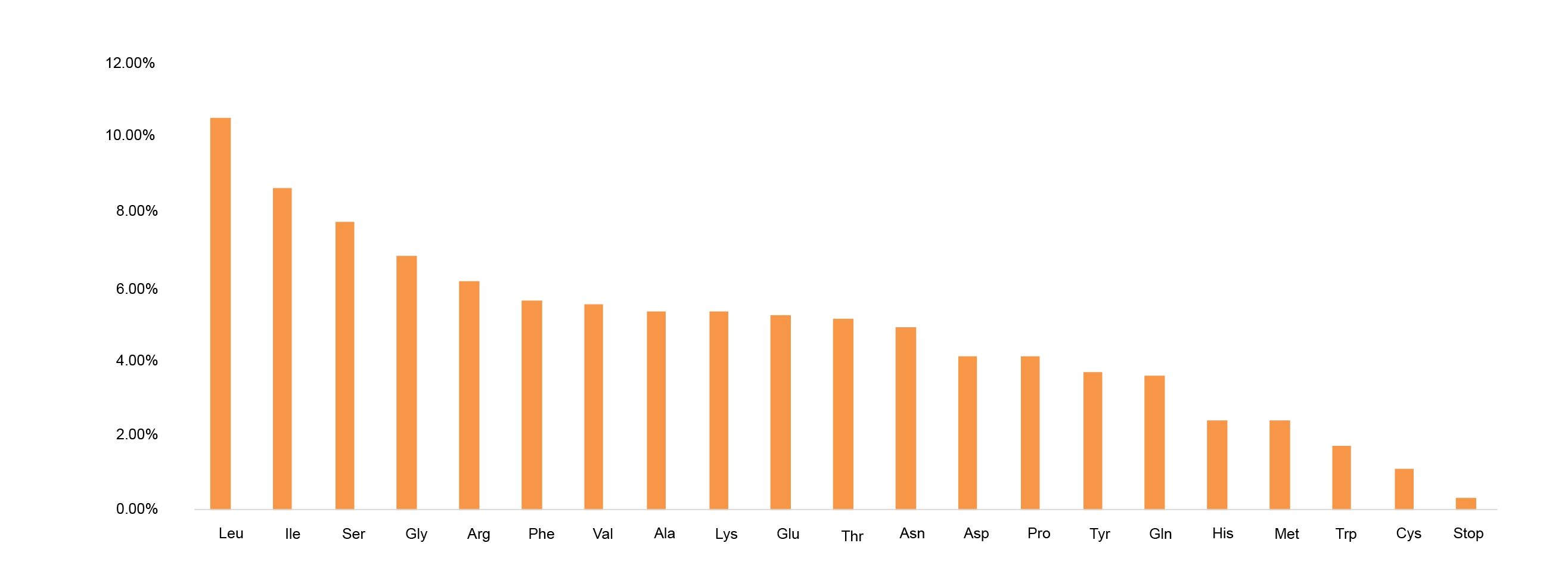

Supplement: Supplementary file 6 [file Image2.JPEG]
