# Supplementary material for: Comparative Genomic and Phylogenetic Analysis of Chloroplast Genomes of Hawthorn (Crataegus spp.) in Southwest China
Source: Front Genet. 2022 Jul 4;13:900357. doi: 10.3389/fgene.2022.900357 (PMC9289535; doi:10.3389/fgene.2022.900357)
Supplement: Supplementary file 4 [file Table8.docx]

Table S8. Morphological analysis of eight hawthorn species native to China according to the Flora of China.

| species | *C. pinnatifida* | *C. pinnatifida* var*. major* | *C. hupehensis* | *C. cuneata* | *C. scabrifolia* | *C. chungtienensis* | *C. oresbia* | *C. kansuensis* |
| --- | --- | --- | --- | --- | --- | --- | --- | --- |
| Type | trees | trees | trees or shrubs | shrubs | trees | shrubs | shrubs | shrubs or trees |
| Thorns | thorns present | thorns present | thorns present | thorns present | thorns absent | thorns present | thorns present | thorns present |
| Leaf shape | broadly ovate or triangular-ovate | broadly ovate or triangular-ovate | ovate or ovate-oblong | broadly obovate or obovate-elliptic | rhombic-ovate | broadly ovate | broadly ovate | broadly ovate |
| Leaf margin serrate | sharply | sharply | cuneate | sharply | obtusely | sharply | sharply | sharply |
| Leaf base | truncate or broadly cuneate | truncate or broadly cuneate | cuneate or subrounded | cuneate or attenuate | cuneate | cuneate | cuneate | truncate or broadly cuneate |
| Leaf lobe | 3–5 pairs of lobes | 3–5 pairs of lobes | 2–4 pairs of lobes | 3–5 pairs of lobes | not lobed | 3–5 pairs of lobes | 3–5 pairs of lobes | 5–7 pairs of lobes |
| Stipules | persistent | persistent | caducous | persistent | caducous | caducous | caducous | caducous |
| Inflorescence | corymb | corymb | corymb | corymb | corymb | corymb | corymb | corymb |
| Peduncle | white tomentose | white tomentose | glabrous | white tomentose | glabrous | glabrous | white tomentose | glabrous |
| Stamens per flower | 20 | 20 | 20 | 20 | 20 | 20 | 20 | 15-20 |
| Pome diameter | 1-2.5 cm | 2.5 cm | 2.5 cm | 1-2 cm | 1.5-2.5 cm | 0.6 cm | 0.6 cm | 0.6 cm |
| Pome color | dark red | dark red | red | red or yellow | yellow | red | reddish | red or yellow |
| Number of pyrenes | 3-5 | 3-5 | 5 | 4-5 | 5 | 1-3 | 2-3 | 2-3 |
| Shape of pyrenes | smooth on both inner sides | smooth on both inner sides | smooth on both inner sides | smooth on both inner sides | smooth on both inner sides | concave scars on both inner sides | concave scars on both inner sides | concave scars on both inner sides |
